# Key Points in Remote-Controlled Drug Delivery: From the Carrier Design to Clinical Trials

**DOI:** 10.3390/ijms22179149

**Published:** 2021-08-24

**Authors:** Denis V. Voronin, Anatolii A. Abalymov, Yulia I. Svenskaya, Maria V. Lomova

**Affiliations:** 1 Science Medical Center, Saratov State University, Astrakhanskaya St. 83, 410012 Saratov, Russia; anatolii.abalymov@ugent.be (A.A.A.); svenskaya@info.sgu.ru (Y.I.S.); lomovamv85@mail.ru (M.V.L.); 2 Department of Physical and Colloid Chemistry, National University of Oil and Gas “Gubkin University”, Leninsky Prospekt 65, 119991 Moscow, Russia

**Keywords:** drug delivery systems, active targeting in vivo, physiological barriers, remote navigation, magnetic fields, electric fields, ultrasound, light-responsive systems, exposure limits, clinical translation

## Abstract

The increased research activity aiming at improved delivery of pharmaceutical molecules indicates the expansion of the field. An efficient therapeutic delivery approach is based on the optimal choice of drug-carrying vehicle, successful targeting, and payload release enabling the site-specific accumulation of the therapeutic molecules. However, designing the formulation endowed with the targeting properties in vitro does not guarantee its selective delivery in vivo. The various biological barriers that the carrier encounters upon intravascular administration should be adequately addressed in its overall design to reduce the off-target effects and unwanted toxicity in vivo and thereby enhance the therapeutic efficacy of the payload. Here, we discuss the main parameters of remote-controlled drug delivery systems: (i) key principles of the carrier selection; (ii) the most significant physiological barriers and limitations associated with the drug delivery; (iii) major concepts for its targeting and cargo release stimulation by external stimuli in vivo. The clinical translation for drug delivery systems is also described along with the main challenges, key parameters, and examples of successfully translated drug delivery platforms. The essential steps on the way from drug delivery system design to clinical trials are summarized, arranged, and discussed.

## 1. Introduction

The core study of new types of drug carriers on living systems remains challenging and requires the involvement and collaboration of diverse high-performing research teams, which are made up of specialists with a wide range of expertise: from natural scientists developing the product to managers introducing it to the market. All these people are united by a common idea, but at the same time, they independently and consistently apply their skills and knowledge in promoting the most effective and low-toxic forms of drugs. The most striking experience of recent years is the coronavirus disease (COVID-19) pandemic. Within the shortest possible time, the authorities, manufacturers, and doctors managed to establish the release production of special drugs (dexamethasone and remdesivir were approved by the US Food and Drug Administration (FDA) in October 2020; baricitinib in combination with remdesivir was approved by the FDA in November 2020; the FDA has granted emergency use authorization for convalescent plasma therapy), adapt treatment methods, and also start the vaccination of the population. This is an example of the rapid approvement of drugs and treatment methods to meet modern challenges and preserve people’s life and health. Among others, the COVID-19 pandemic reveals the need for continuous adjustment of the requirements for novel drug formulations and their dosage forms.

The demand for novel pharmaceutical formulations and their controllable and targeted delivery is related to the multitude of side effects, which arise from the out-of-date treatment protocols regarding the systemic and iterative administration of drugs. In conventional drug formulations, the bioactive molecules may display low bioavailability, poor water solubility, and biological degradation [1]. Furthermore, the emerging novel types of pharmaceutics, which include nucleic acids, peptides, proteins, and cells, require specific ways of delivery to maintain their bioactivity, reduce immunogenicity, and improve targeting selectivity. The employment of the drug delivery systems is believed to be one of the “core paradigms” to overcome these challenges [2]. Therefore, the future development and evolution of pharmaceutical therapy are closely associated with drug delivery strategies.

Intravenous administration appears as a promising strategy for drug delivery. The blood vessels are a broad avenue for direct access to internal organs avoiding skin, mucosal, or gastrointestinal tract barriers. This is of great importance in the therapy of various cancers and cardiovascular diseases. However, compared to direct intratumoral injections, intravenous administration lacks the locality, which may result in an undesired accumulation of drug delivery systems (DDS) in the side organs. To overcome this, various targeting approaches are applied, including physical, chemical, and biological addressing of DDS through the embedding of stimuli-responsive components, targeting ligands, or vectors into the DDS structure. Additionally, to increase the DDS localization in the target organs, the modification of the DDS structure can be attended with specific injection ways such as endovascular injection directly to vessels supplying the organ [3].

This review highlights the essential steps of delivery system design including (i) the choice of the drug carrier with the defined properties for the desired drug; (ii) the definition of the physiological barriers and the other obstacles that impede therapeutic or theranostics efficacy; (iii) the selection of the way for remote navigation and triggering the drug release; and (iv) the ways of introduction of the newly developed drug formulation for clinical use. Finally, we have concluded the review with a scheme with the step-by-step implementation of a drug delivery system from the design to possible clinical trials. We believe that the unified approach for the development of drug delivery systems will provide fundamentally new forms of drugs with a wide range of properties and accelerate their clinical employment.

## 2. Selection of the Drug Carriers Depending on the Type of Encapsulating Substances

It has been repeatedly emphasized that the primary objectives of the targeted drug delivery are to reduce the therapeutic dose of highly toxic drugs and to minimize the possible side effects [4]. With this respect, the most important prerequisites for the development of novel drug formulations for targeted delivery are maintaining the maximal activity of the drugs during and after the preparation of delivery systems as well as minimizing their impact on the environment. Additionally, the drug delivery system should come with multifunctionality, which is provided by combining in a single carrier various functional molecules, nanoparticles, and enzymes to respond to passive and/or active targeting, external drug release triggering, and ensure the reliable coupling and high loading capacity of the delivered drugs [5].

**Figure 1:**
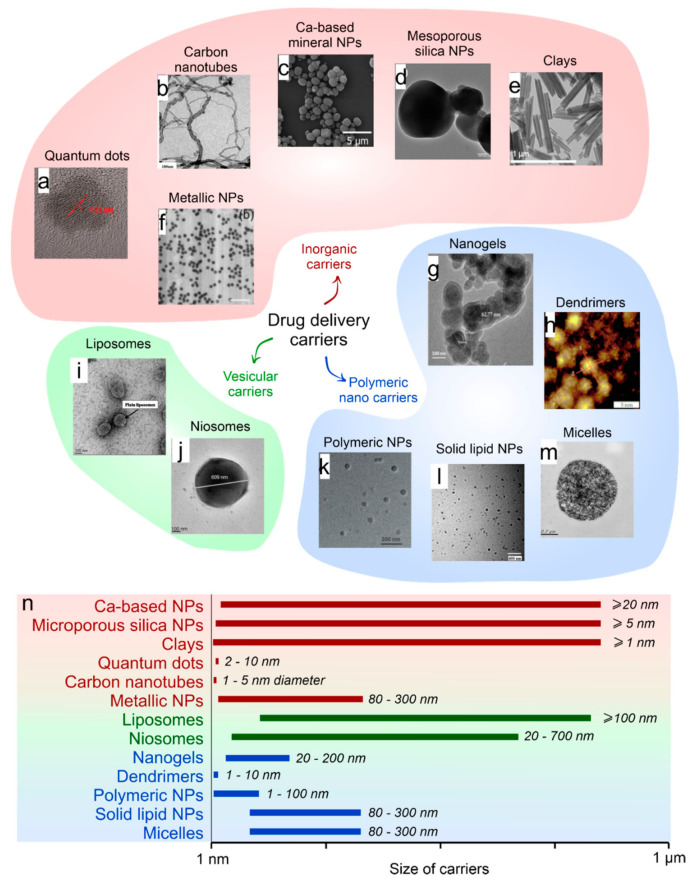
(**a**) Classification of the most commonly used nanocarriers for drug delivery (**a**–**m**) with included photos of the quantum dots (SEM image reprinted with permission from Frontiers [18]). (**b**) CNTs (SEM image reprinted with permission from Frontiers [19]). (**c**) Ca-based minerals (SEM image reprinted with permission from ACS [20]). (**d**) Mesoporous silica (SEM image reprinted with permission from ACS [21]). (**e**) Clays (SEM image reprinted with permission from Frontiers [22]). (**f**) Metallic (SEM image reprinted with permission from Elsevier [23]). (**g**) Nanogels (SEM image reprinted with permission from Frontiers [23]). (**h**) Dendrimers (AFM image reprinted with permission from Hindawi [24]). (**i**) Liposomes (TEM image reprinted with permission from Frontiers [25]). (**j**) Niosomes (TEM image reprinted with permission from Elsevier [26]). (**k**) Polymeric nanoparticles (TEM image reprinted with permission from Frontiers [27]). (**l**) Solid lipid NPs (TEM image reprinted with permission from Hindawi [28]). (**m**) Micelles (TEM image reprinted with permission from Frontiers [29]). (**n**) Size distribution of drug delivery carriers.

The development of drug delivery systems should include the choice of the appropriate drug carrier type, the drug to deliver, and its loading technique [6,7,8,9]. Currently, the drug carriers include the diversity of micro- and nanosized polymeric capsules, liposomes, hard-core particles, and polymersomes [8,10,11,12,13,14] (Figure 1). All of them have their advantages and disadvantages; however, they gradually find application in various fields of biomedicine. Liposomes represent one of the most clinically established technologies applied for the encapsulation and delivery of a tremendous diversity of pharmaceuticals incorporated within their hydrophilic and hydrophobic compartments [15]. Control over the properties of such carriers can be achieved by varying the type of lipids used in their preparation. However, the wide range of challenges in biomedicine push one to take a step beyond liposomal drug delivery [16,17].

The localization of drugs delivered by the drug carriers can be achieved in two ways: (i) by localizing the carrier after its systemic administration via specific molecular interactions and/or remote navigation by external fields; (ii) by the local administration including intratumoral, endovascular, or direct administration to the target organs and tissues. In turn, the success of the site-specific therapy is directly associated with the efficiency of loading and release of the drug along with the biocompatibility, non-immunogenicity, and biodegradability of the delivery carrier [30].

In cancer-targeted therapy, the drug accumulation at the tumor site may be additionally promoted due to the enhanced permeability and retention (EPR) effect. It is hypothesized that the aberrant angiogenesis of tumor neovasculature results in abnormal fluid transport dynamics. This leads to an improved accumulation of submicron DDS and macromolecular drugs in cancer tissue compared to normal tissue [31]. The efficiency of the EPR effect was demonstrated on macromolecular complexes, proteins, and polymers of various molecular weights, liposomes, nanoparticles, lipids, including those conjugated with commercially available drugs [32,33]. However, the reliable drug delivery employing only the EPR effect is challenging due to its sufficient heterogeneity depending on the tumor nature and microenvironment. Therefore, the DDS accumulation may vary considerably from patient to patient [33].

The study of drug carriers in vitro is one of the first stages of testing new types of drug formulations. Standard and widespread in vitro protocols are required to characterize the physicochemical (stability, release profile, loading efficiency) and cytotoxic properties of drug carriers. The conclusions based on these studies determine the areas of further application and the nature of the tasks solved in vitro/in vivo using these carriers (tissue engineering/regeneration, 3D cell printing, 3D spheroid generation, test platform creation).

Furthermore, the elaborated drug delivery system should be evaluated in vivo using an appropriate animal model, as a great number of carriers fail during the in vitro to in vivo translation.

In Table 1, we have summarized some recent examples of targeted delivery systems studied in vivo along with the relationship between the types of active drugs, their formulations, the mode of delivery to the target organ, and the drug release mechanism upon administration to the blood vessels. In this regard, we have considered the most striking examples of delivery systems of recent years for anticancer drugs, nucleic acids, growth factors, etc. Four factors were important parameters for the choice of studies: the structure of the carrier, the possibility of targeting, the control of the release, and in vivo evaluation of treatment protocols employing the developed DDS.

Analyzing the examples presented in Table 1, one can outline the following points: Various types of delivery systems, which are different in their properties, can be employed to deliver and localize the same type of drug. By this means, the choice of the drug type should be the starting point in the preparation of the targeted delivery system. The physicochemical properties of drugs (solubility, partition coefficient, hydrogen bonding, complexation, bioisosterism) are the first thing to pay attention to. For example, a completely different strategy should be applied for the loading of hydrophilic and hydrophobic drugs into the same carrier [58,59,60].

The research experience accumulated to date allows for predicting the behavior of various types of drug carriers in experiments in vivo. As a result, a particular type of drug carrier with the desired properties can be chosen before the drug loading depending on its suggested application. A striking example is a difference in pH between normal cells and cancerous cells. The development of carriers degradable in an acidic environment, such as mineral carriers (calcium carbonates and phosphates), has attracted much attention in the last decade [61,62]. In some cases, the carrier may be one of the primary parts that the organ can use to repair damaged tissue [20].

The experience of the successful drug loading to the particular type of carrier can be translated to another drug, which has a similar chemical structure [8,63]. However, it should be taken into consideration that encapsulation efficiency might be different in this case, as it was shown for doxorubicin, mitomycin C, camptothecin, methotrexate, verapamil, and 9AC drugs immobilizing into carbon–iron carriers [64]. On the other hand, one drug can interact with the carriers in many ways; this property allows for using various carriers that will be equally efficient at encapsulating the same molecule [60,65,66,67,68,69].

Basing on the data on delivery and release, it is possible to find out the most optimal type of carriers to achieve the maximum therapeutic effect. In bone tissue engineering, it might be beneficial to use CaCO_3_ or Ca_3_(PO_4_)_2_ micro- or nanoparticles, which can simultaneously act as carriers of various bioactive molecules and a source of calcium and phosphate ions [20,70]. Dual and multiple drug-loaded carriers are widely used in anticancer treatment. Furthermore, the application of carriers capable of multimodal drug delivery represents an important approach to combinatory treatment as providing a synergistic effect [71]. Thus, for example, magnetic carriers loaded with an anticancer drug provide both drug targeting to tumor and hyperthermia functions [72].

## 3. Barriers and Limitations Associated with Targeted Drug Delivery

Each delivery route has its limitations associated with certain biological barriers. These barriers prevent the successful accumulation of the drug-loaded carriers at the diseased sites, limiting their bioavailability and therapeutic potential.

**Figure 2:**
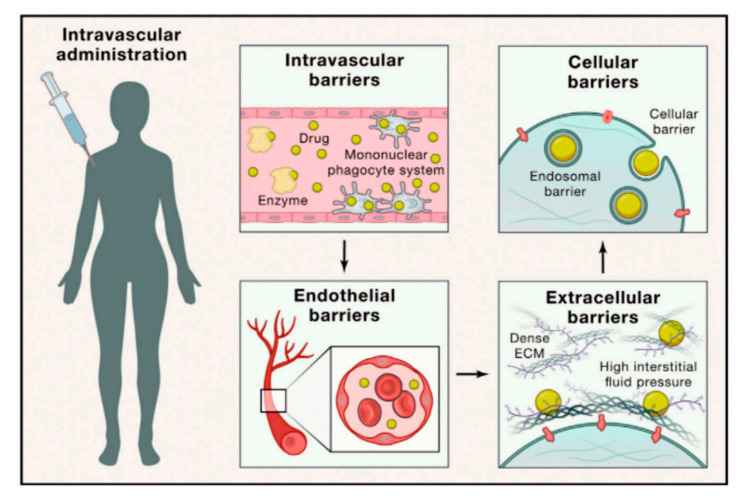
Biological barriers for site-specific drug delivery. Reprinted with permission from ^16^© 2021 Published by Elsevier Inc. License number 4997111241312.

The systemically delivered therapeutics encounter serial biological barriers including intravascular barriers, endothelial barriers, extracellular barriers, and cellular barriers (Figure 2) [16]. By this means, the administered drug-carrying platforms face such obstacles as (i) opsonization followed with subsequent sequestration by mononuclear phagocyte system, (ii) hemorheological/blood vessel flow limitations, (iii) pressure gradients, and (iv) cellular internalization including endosomal compartmentalization [73].

The major limitation of nanotherapeutic delivery is associated with nonspecific uptake in healthy organs. The injected carriers undergo opsonization and subsequent uptake by resident macrophages of the mononuclear phagocyte system, resulting in a high accumulation of this formulation in the spleen and the liver [74]. The opsonization of carriers involves the coating of their surface by such plasma proteins as serum albumin, apolipoproteins, and immunoglobulins [75]. The surface charge of the carriers plays an important role in protein adsorption, which in turn affects their pharmacokinetics and biodistribution. It was demonstrated that highly cationic as well as highly anionic nanoparticles are rapidly cleared from circulation [76]. Meanwhile, neutral ones, as well as those with a slight charge, show significantly prolonged circulating half-lives [77].

Rapid corona formation is found to affect hemolysis, thrombocyte activation, nanoparticle uptake, and endothelial cell death at an early exposure time [75]. The nature of the proteins in the corona is determined by the local chemical property of the nanomaterial. However, even for the particles of identical materials, both size and surface properties were found to play a very significant role in determining the biologically active proteins in the nanoparticle coronas, affecting the biological impacts [78]. These parameters critically determine the protein binding quantitatively but not qualitatively [75,79].

Nanoparticle fluid dynamics in blood vessels is highly dependent on the size and geometry of the construct, and in turn, it affects the margination dynamics of the carriers to vascular walls [73]. The lateral drift of the carriers to endothelial walls (margination dynamics) should be taken into consideration while designing the delivery system. Association with vessel walls favors particle–cell binding and receptor-ligand interactions in active targeting strategies and enables extravasation through the fenestrated vasculature of tumors [73]. It was suggested that the particles used as drug delivery systems should have a radius smaller than 100 nm to facilitate margination and interaction with the endothelium [80]. Whereas the particles used as nanoharvesting agents (e.g., in proteomics or genomics analysis) should have a radius close to this value to minimize margination and increase their circulation time. At the same time, it is known that the permeabilized vasculature of tumors may vary from 200 to 800 nm [32]. Thus, such a high permeability of the tumor vasculature compared to normal tissue could allow particles even larger than 100 nm to enter the tumor interstitial space. Concerning the carrier shape, it was said that spherical particles of small size migrated in a cell-free layer, at a considerable distance from endothelial surfaces [73]. That limits both active targeting strategies and effective accumulation through passive targeting mechanisms. Meanwhile, nonspherical particles under flow exhibit tumbling and rolling dynamics; thus, they are capable of oscillating from one wall to the opposite wall in a vessel that can increase their margination [73,81].

Interaction of the carriers with the vascular wall depends on their surface charge as well [76]. Neutral particles can escape such an interaction. Meanwhile, the charged ones interact with the vascular wall through electrical interactions.

In cancer therapy, substantial barriers occur for nanoparticle accumulation in tumors [82]. The first obstacle is associated with a high intratumoral pressure resulting from interrupted vasculature, the aggressive nature of cellular growth, impaired lymphatics, and dense extracellular matrix [73,83]. The tumor vasculature is highly abnormal, exhibiting an uneven distribution with zones of both increased and sparse vascular density, hierarchical disorganization, serpentine structure, and irregular branching [84]. Such heterogeneous vasculature represents another substantial limitation to the intratumoral accumulation of the particles and restricts the permeability of some tumor sites. Furthermore, the permeability of tumor vessels differs in patients and leads to the difference in tumor targeting and as a result in various therapeutic efficacy [85].

Furthermore, the carriers have to undergo cellular membrane transversal and endosomal compartmentalization to release the cargo, exerting therapeutic effects on cytoplasmic and nuclear targets [73]. The size, shape, and surface charge of the carriers affect their internalization [86]. It should be also noted that endosomal compartmentalization of internalized carriers, subjected to a low pH environment and enzymes, proves detrimental to cargo, especially to genetic material fate (e.g., m- and siRNAs or plasmid DNA) [73]. In light of the highly degradative endosomal environment, numerous studies have focused on the development of various endosomal escape strategies to be applied in drug delivery depending on the carrier type and targeted organ/cell [73,87,88]. The use of cationic polymers, cell-penetrating peptides, photo- or pH-sensitive compounds in the carrier design has been considered effective to induce the release of payload therapeutics from endosomal compartments when it is necessary [89,90,91,92].

The stability of the cargo is another important issue limiting drug delivery. Genetic material shows low stability in biological media as well that results in serious limitations for its applicability in physiological environments [93]. Delivering pharmacologically active proteins/peptides to specific tissues also faces their instability during blood circulation, degradation by enzymes, short half-life, immunogenicity, and inability to cross cell membranes [94,95]. The encapsulation of various proteins and peptides is challenging itself, especially concerning certain carrier types as may affect the payload activity [96].

All the listed biological barriers that a carrier encounters upon intravascular administration should be adequately addressed in its overall design. Thus, the effective particulate delivery system should escape immune clearance in the liver and spleen, permeate across the endothelium into target tissues, and then penetrate through the tissue interstitium. Furthermore, it should be endocytosed by target cells, diffuse through the cytoplasm, and eventually enter into the nucleus, if required [97]. The delivery system should not only promote targeted transportation and control the release of such sensitive cargo in targeted sites but also protect them from degradation. Moreover, a successful delivery platform should also take into consideration the disease type and state of its progression, as these parameters sufficiently affect these barriers [73].

## 4. Physical Addressing and Release of Encapsulated Drugs by External Stimuli In Vivo. Principles and Safety Considerations

### 4.1. Remote Navigation and Triggered Release Mediated by the Magnetic Field

The magnetic field is attractive for remote targeting of delivery systems in vivo due to several reasons. First, constant exposure to the geomagnetic field is a natural state of all living organisms on the Earth. The magnetic field is non-ionizing; therefore, it is generally considered harmless itself. Additionally, the magnetic field has a good permeability into the tissues [5]. This gives rise to employing the magnetic field in medical applications including diagnostics and therapy.

The targeting can be achieved by the local exposure of the desired area by a non-uniform static magnetic field. The principle of magnetic targeting is the formation of local inhomogeneity in the density of magnetic field lines (i.e., the spatial magnetic field gradient). The magnetically responsive carrier will move to the area with the highest density of the lines. Ordinarily, all biological and organic materials are diamagnetic with low magnetic susceptibility and negative magnetization response [98,99]. Therefore, the delivery carriers modified with magnetic particles possessing a positive magnetization response and high magnetic susceptibility can be selectively localized by the non-uniform magnetic field [100].

Although static magnetic fields are considered safe, there are three established mechanisms through which static magnetic fields can interact with the living matter according to the International Commission on Non-Ionizing Radiation Protection (ICNIRP) [101]. These include magnetic induction, magneto-mechanical, and electronic interactions.

Magnetic induction implies the effect of static magnetic fields on moving objects, resulting in the induction of electric currents. For instance, the magnetic field may affect the flowing blood-inducing current affecting the heart rate [102]. The movement of the whole body along the gradient of the magnetic field may induce the electrical current, affecting nerve stimulations. Additionally, the World Health Organization (WHO) reported that movement in a magnetic field gradient may induce the sensation of vertigo and nausea if the field exceeds about 2–4 T [103].

Magneto-mechanical interactions imply the orientation of materials and induction of magneto-mechanical translations in the magnetic field gradient. Potentially, this may result in the reorientation of body tissues and affect the systemic blood flow in strong magnetic fields (>17 T), yet, practically, the effect of the magnetic field is negligible due to the low magnetic susceptibility of biological tissues [101]. Finally, strong static magnetic fields may affect the rate of metabolic reactions. The typical example is hemoglobin oxygenation [104,105].

Thus, safe organism exposure is available up to a particular induction value of the static magnetic field. The FDA declared that the static magnetic field of 2 T is safe for whole-body exposure in clinical use [106]. However, for the exposure up to 5 T, the patient should be monitored for symptoms referred to the nervous system according to the International Radiation Protection Association (IRPA) [107]. In turn, ICNIRP Guidelines on Limits of Exposure to Static Magnetic Fields (2009) states the limit of exposure of the head and trunk is 2 T and that of limbs is 8 T in a controlled environment, restricting the body movement [101]. The whole body can be safely exposed up to 4 T under the same conditions [108]. However, acute exposure of the general public should not exceed 400 mT in everyday life to prevent the harmful effect of the implanted electronic medical devices and metallic implants [109].

Unlike targeting, the magnetic field-mediated release is based on the alternating magnetic field (AMF). This makes the simultaneous use of static and alternating magnetic fields promising for the targeting and release of encapsulated drugs in one single setup. Moreover, the devices combining the gradient of static magnetic field and AMF are widely employed in clinical practice for magnetic resonance imaging (MRI) diagnostics.

**Figure 3:**
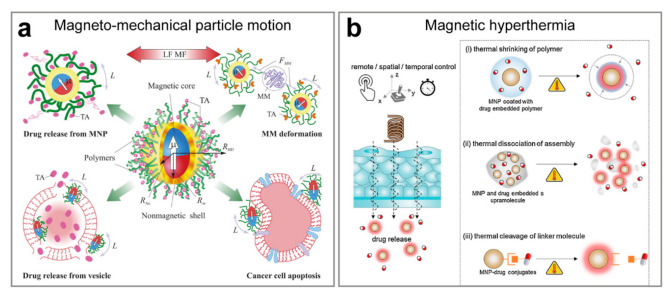
Drug release is triggered by an alternating magnetic field. (**a**) Schematic presentation of currently explored directions in nanomedicine and drug delivery that exploit magneto-mechanical actuation of functionalized MNPs in a low-frequency magnetic field. Abbreviations correspond to a low-frequency AMF (LF MF), therapeutic agent (TA), the magnetic moment of (μ), the torque applied to MNP (L), the macromolecule (e.g., enzyme) attached to MNP (MM), and the magneto-mechanical force causing macromolecule deformation (*F_MM_*). A schematic of functionalized MNP is presented having a superparamagnetic core of a radius *R_m_*, a solid shell (e.g., gold) of a radius *R_Au_,* and water-soluble polymeric corona. The hydrodynamic radius of the functionalized MNP is *R_HD_*. Reproduced with permission from [112]. Copyright 2015, Elsevier B.V. (**b**) Magnetothermally triggered drug delivery systems. Due to the remote and spatiotemporal controllability of AMF, drugs can be released and delivered at a target at the desired time. Various types of drug release systems are possible, based on (i) shrinkage of thermoresponsive polymers, (ii) thermal dissociation of self-assembled nanostructures, and (iii) thermal cleavage of temperature-sensitive linker molecules. Reproduced with permission from [111]. Copyright 2017, Elsevier Ltd.

There are two principal ways of how the AMF may trigger the release from the magnetically responsive carrier (Figure 3). The necessary condition is that the carrier has to be modified with single-domain superparamagnetic nanoparticles. The release may be induced by the mechanical motion of magnetic particles [110] or by magnetic hyperthermia [111].

**Figure 4:**
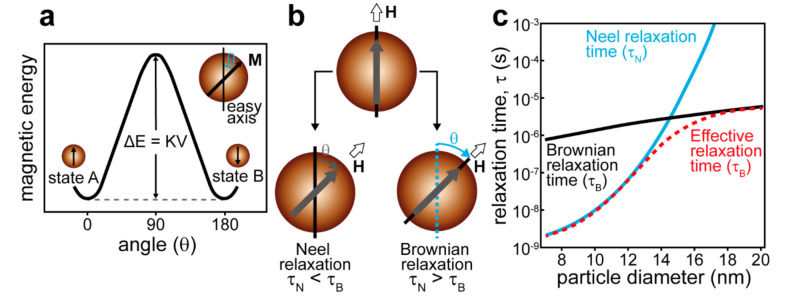
(**a**) The magnetic anisotropy energy barrier (ΔE) as a function of the angle between the easy axis and the magnetization orientation in a single magnetic domain regime. (**b**) Schematic presentation of the magnetic loss from MNPs through Neel and Brownian relaxation. (i) Neel relaxation: magnetic spins rotate while the particles remain fixed. (ii) Brownian relaxation: magnetic spins remain fixed along the crystalline axis while the particles physically rotate. (**c**) Relaxation times as a function of the particle diameter for single-domain magnetite MNPs. The relaxation times are given by Equations (1)–(3). Adapted with permission from [111]. Copyright 2017, Elsevier Ltd.

The mechanical or thermal effects of AMF are defined by the dominating mechanism of magnetic moment relaxation, which depends on the particle size, magnetic crystalline anisotropy, surrounding medium, and applied AFM frequency. For a single-domain nanoparticle, the magnetic moment is aligned with the energetically favorable direction of the spontaneous magnetization (easy axis) with the two opposite directions available (Figure 4a). These two positions are separated by the magnetocrystalline anisotropy barrier. In case the magnetic field energy is higher than the anisotropy barrier, the magnetic moment is flipped and aligned in the opposite direction. The excess energy of the magnetic field is released as heat. This is the Neel relaxation (Figure 4b). The characteristic time of Neel relaxation is given by the ratio of the anisotropy energy *KV* and thermal energy *k_B_T*

(1) $$\tau_{N}=\tau_{0}\exp\left(\frac{KV}{k_{B}T}\right)$$

where *τ_0_* is the characteristic attempt time (10^−9^–10^−10^ s), *K* is the magnetic anisotropy constant, *V* is the particle volume, *k_B_* is the Boltzmann constant, and *T* is the temperature [113].

If the particle size exceeds some critical value, the magnetocrystalline anisotropy barrier will hinder the Neel relaxation, which will result in the movement of the whole particle following the reorientation of the magnetic moment fixed toward the easy axis [114]. The magnetic field energy will dissipate as heat due to viscous friction between the particle and surrounding medium (Figure 4b). This is the Brown relaxation with the characteristic time given by

(2) $$\tau_{B}=\frac{4\pi \eta r_{h}^{3}}{k_{B}T}$$

where *η* is the medium viscosity, *r_h_* is the hydrodynamic radius of the particle, *k_B_* is the Boltzmann constant, and *T* is the temperature [113].

The effective relaxation time for superparamagnetic nanoparticles is given by

(3) $$\tau_{eff}=\frac{\tau_{N}\tau_{B}}{\tau_{N}+\tau_{B}}$$

Figure 4c shows the relative contribution of Neel and Brown relaxation in the effective relaxation time depending on the particle size [111]. To ensure the energy of the magnetic field is converted to mechanical motion, the particle has to be larger than some critical size. However, for a low-frequency AMF, when the half-period of field oscillation exceeds the Brown relaxation time, the smaller particles can also follow the magnetic moment motion [112].

Thus, the selective remote actuation of the drug carriers by AMF required the proper field amplitude and frequency to keep the body tissues intact. The Institute of Electrical and Electronics Engineers (IEEE) introduced the following gradation of AMFs depending on the frequency range: the low-frequency magnetic field is the field with a frequency < 300 kHz, and the high-frequency field has a frequency >3 MHz [115]. In 1998, the ICNIRP limits the product of magnetic flux × field frequency to 25 mT × Hz^−1^ for a frequency range from tens to hundreds of Hz and 2000 mT × Hz^−1^ for the range of hundreds of kHz at workplaces, whereas the limits in the same frequency bands for the general population are 5 mT × Hz^−1^ and 920 mT × Hz^−1^, respectively [116]. Furthermore, the IEEE sets the limits of AFM exposure to prevent painful tissue stimulation in the frequency range from 0 to 5 MHz, tissue overheating in the range from 100 kHz to 300 GHz, and both of these in the intermediate range from 100 kHz to 5 MHz. The particular exposure reference levels (ERL) depending on the frequency, magnetic flux density, magnetic field strength, body part, and exposure mode are given in the corresponding IEEE standard issued in 2019 [115]. Golovin et al. have given an illustrative and comprehensive summary of natural and technogenic AMF and exposure limits concerning the ICNIRP guidelines (1998) in their review [117].

In biomedicine, the main type of AMF interaction with the organism is the Faraday induction of electric fields and associated currents in the tissues. In 1988, Brezovich experimentally determined the limit criterion of the product magnetic field strength (*H*) × frequency (*f*) for hyperthermia treatment as 4.85 × 10^8^ A/m·s to prevent the tissue overheating due to eddy current losses [118]. The contribution of AMF-induced electric fields to heat generation is measured with the specific absorption rate (SAR) of the tissues. The latest ICNIRP basic restrictions limit the local SAR in human exposure to 2–10 W/kg, depending on the body part [115]. According to Golovin’s estimations, at a field frequency of 10 kHz, the tissue SAR is about 1 W/kg, which corresponds to the temperature increase of 1 K. Thus, the AMF with the frequency ≤10 kHz can be considered as non-heating, while the AMF with the frequency ≥100 kHz can be considered as heating within the range of magnetic field amplitudes typical for biomedical applications.

To sum up this section, we refer to the recent examples of magnetically responsive DDS illustrating various options in cancer therapy employing systemic (intravenous) DDS administration. Liu et al. demonstrated a novel approach for extra deep penetration of magnetically responsive DDS driven by the low-frequency non-heating AMF and following drug release under high-frequency heating AMF [119]. Their delivery system is based on 50 nm ferrimagnetic vortex iron oxide nanorings modified with thermoresponsive polyethyleneimine terminated with isobutyramide groups and conjugated with DOX. In experiments in vivo on the BALB/c nude mice bearing MCF-7/ADR tumors, the nanorings were injected intravenously, and after 6 h of circulation, the tumor site was exposed by non-heating 0.1 kHz AMF for 10 min. The nanorings demonstrated deep penetration and uniform distribution inside the tumor tissue, which is evidence of the successful penetration through the stromal barrier. Upon penetration, the tumors were treated with heating AMF (360 kHz, 24 kA/m, 10 min) to trigger the DOX release. As the main outcome of this study, the sequential exposure to low-frequency and high-frequency magnetic fields was shown to be the most efficient to reach 86.2% of DOX delivery into the cancer cell nucleus, which is the result of DDS design and the proper order of the AMF treatment. Furthermore, this approach was successfully applied to MDA-MB-231 breast tumor and BxPC-3 pancreatic tumor models in vivo.

Shen et al. reported on the magnetic “nanoraspberry” clusters coated with an exosome shell [120]. Additionally, the clusters were loaded with DOX. It was shown that the capsules injected into the blood flow can effectively accumulate into GFP-B16F10 lung metastases due to the exosome-derived margination effect. The DDS is responsive to the magnetic field, and therefore, the drug release can be triggered by external AMF exposure (50 kHz, 4 kA/m). Owing to a high density, the clusters demonstrated deep tumor penetration and derived nanoparticle-induced extracellular leakiness. This resulted in the disruption of tumor tissue, which further promoted the penetration of therapeutic agents and cytotoxic T cells. Furthermore, the application of AMF was shown to enhance this effect.

Liu et al. designed magnetically responsive DDS based on the Mn-Zn ferrite clusters with encapsulated paclitaxel (PTX) covered with biocompatible DSPE-PEG2000 phospholipid and modified with tripeptide of arginine-glycine-aspartic acid (RGD), which can specifically couple with the proteins expressed on the tumor neovascular epithelial cells [121]. The resulted DDS system was shown to be an effective theranostics agent combining diagnostics modality via MRI and thermal imaging along with therapeutics modality via thermo-responsive release of PTX under heating AMF (390 kHz, 2.58 kA/m) exposure. In experiments in vivo on the breast cancer 4T1 bearing BALB/c mice, it was shown that this DDS provides an enchased penetration ability into solid tumor tissue when intravenous injection is attended with AFM exposure of the tumor site, and the most therapeutic effect was reached in a combination of chemotherapy and magnetic hyperthermia treatment.

### 4.2. Enhancement of Site-Specific Drug Delivery with Ultrasound

**Figure 5:**
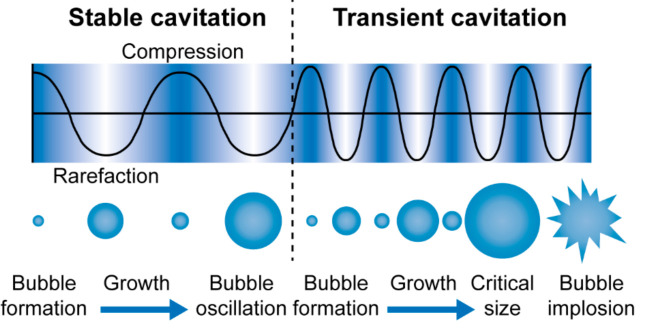
Schematic representation of cavitation bubbles displaying stable and transient cavitation due to continuous compression and rarefaction of the liquid medium under the propagation of an ultrasound wave. Adapted with permission from [126]. Published by The Royal Society of Chemistry.

Along with magnetic fields, ultrasound is another physical phenomenon that is widely employed in medicine for diagnostic and therapeutic applications. The term ultrasound implies the acoustic waves with a frequency higher than 20 kHz, which cannot be heard by the human ear [122]. Ultrasound is non-ionizing and has a good penetration in body tissue [123]. Although ultrasound cannot be used for the external navigation of delivery systems directly as a magnetic field can, it can improve the site-specific infiltration of systemically administrated drugs and trigger the release from responsive delivery carriers due to local ultrasound exposure [124]. The driving force of this is the acoustic cavitation that is the formation, growth, and oscillation (in case of stable cavitation) or collapse (in case of transient cavitation) of gas bubbles due to continuous compression and rarefaction of the liquid medium under the ultrasound wave propagation [125] (Figure 5).

**Figure 6:**
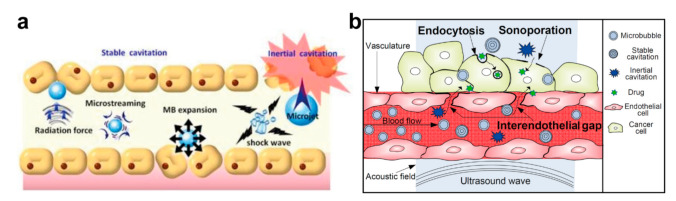
(**a**) Physical mechanisms of stable and inertial cavitation underlying the enhanced vessel permeability and triggering the drug release. Reproduced with permission from [133]. Copyright 2021, Ivyspring International Publisher. (**b**) Overview of the three main passive and active delivery routes enhanced by ultrasound-driven microbubbles. Sonoporation, which refers to transient and reversible membrane perforation by acoustic cavitation, allows macromolecules to passively diffuse into the cell. The interendothelial gap, which refers to the alteration of vascular integrity and the opening of the interendothelial junction by acoustic cavitation, provides an active route for macromolecule delivery into the extravascular tissues. Endocytosis enhanced by stable cavitation can actively deliver the macromolecules into the cell via vesicles. Reproduced with permission from [131]. Copyright 2018, Elsevier B.V.

From the mechanical point of view, the oscillation and collapse of cavitation bubbles result in the motion of the surrounding liquid. This leads to the formation of microstreams inducing the shear stress on the immediate objects. Additionally, the violent collapse of cavitation bubbles produces shock waves and fluid microjets [127,128] (Figure 6a). These cavitation-induced phenomena may result in a significant mechanical impact on drug delivery systems, which induces the release of the loaded drug, on the one hand, and improves, for instance, the tumor-site drug penetration, on the other hand, [129]. Additionally, the collapse of cavitation bubbles leads to sonoporation enhancing the vascular and cell membrane permeability (endocytosis) [130,131] (Figure 6b). Finally, these effects can be spatially and temporally controlled due to the non-invasive and local nature of ultrasound treatment [132].

According to WHO classification, the ultrasound may interact with the matter by thermal, cavitation, or mechanical stress mechanism [134]. The thermal mechanism is related to the transformation of acoustic energy to heat with ultrasound absorption. The cavitation mechanism leads to the formation of oscillating or collapsing bubbles due to medium rarefaction under acoustic wave propagation. Mechanical stress occurs due to the generation of shear waves and microjets when the cavitation bubbles are collapsing. Unlike ionization radiation, where the interaction mechanism does not depend on the exposure rate, the dominant mechanism of ultrasound interaction is defined by its intensity, frequency, and exposure conditions [134].

The diagnostic and therapeutic modalities of ultrasound imply different ultrasound intensities and, therefore, different safety regulations. The diagnostic ultrasound is intended to collect information about the tissues and organs without invasion. Thus, it requires a low intensity of exposure. On the other hand, the therapeutic ultrasound has to manipulate tissues, which supposes higher intensities. In 1982, the WHO denoted the typical frequencies and intensities for therapeutic and diagnostic ultrasound [134]. For therapeutic ultrasound, the intensity varied from 100 to 3000 mW/cm^2^ at 1 MHz, whereas the intensity range of diagnostic ultrasound was 1–20 mW/cm^2^ at 2.25 MHz. Furthermore, in 2004, the FDA limited the acoustic output of diagnostic devices (spatial-peak temporal-average intensity) to 720 mW/cm^2^ for all operation modes [135]. Currently, according to the FDA, the diagnostic devices in the US must operate in a 1–20 MHz frequency range with the peak rarefactional pressure of 0–7 MPa, 1–100 cycles in a pulse, and pulse repetition frequency of 100 Hz to 20 kHz [136]. For therapeutic ultrasound, the frequency range of FDA-approved medical devices varies from 20 kHz to 7.5 MHz depending on application and exposure mode [137]. In turn, the Canadian government regulates the spatial-peak temporal-average intensity of the therapeutic ultrasound, which shall not exceed 3 W/cm^2^ [138].

To conclude this section, we refer to the recent examples of cancer therapy employing intravenous administration of DDS attended with ultrasound treatment. These illustrate various strategies of how ultrasound can be employed to trigger the drug release and promote drug penetration via sonoporation and ultrasound-induced cavitation in experiments in vivo. Li et al. described the preparation of yellow-fluorescent carbon dots and poly(amidoamine) dendrimer dual-drug loaded with polyethylene glycol 1000 vitamin E succinate (TPGS) and DOX conjugates for ultrasound-assisted theranostics of multidrug resistance (MDR) tumors [139]. In experiments in vivo on the MCF-7/ADR tumor-bearing nude mice, it was shown that the intravenous injection of dual-drug complexes allows for overcoming the MDR of cancer cells through reducing the intracellular ATP level and mitochondria membrane potential and enhancing the ROS generation. The effect of targeted drug delivery was further enhanced by additional ultrasound treatment (1 MHz, 0.4 W/cm^2^) of the tumor site through the produced sonoporation effect.

Liu et al. developed the DDS with the possibility of burst DOX release triggered by focused ultrasound and followed by the sustained release after ultrasound treatment of the tumor [140]. The DDS is based on the hollow dendritic mesoporous organosilica nanoparticles loaded with DOX, Fe nanoparticles, and thermo-responsive L-menthol. The inclusion of Fe nanoparticles allows for monitoring of the DDS distribution with MRI. Experiments in vivo were carried out on the 4T1 tumor-bearing BALB/c nude mice. The carriers were injected into the tail vein and were accumulated in the tumor in 1 h, which was established with MRI visualization. Furthermore, the tumor site was treated with focused ultrasound to reach the heating level of 45 °C maintained for 800 s. This treatment resulted in the most significant reduction of the tumor volume in comparison with other groups of animals.

Chen et al. proposed DOX-loaded nano-micelles to treat triple-negative breast cancer [141]. The micelles were prepared with PLGA-PEG and modified with anti-epidermal growth factor receptor protein for tumor targeting. The ultrasound (0.5 W/cm^2^, duty cycle: 50%, 5 min)-mediated cavitation was employed to maximize the intratumoral blood perfusion. The micelles were intravenously injected into MDA-MB-468 tumor-bearing mice. It was established that combined vector-targeted delivery attended with ultrasound tumor-site treatment resulted in the most significant tumor-growth inhibition value (about 72%) and led to the survival of the treated animals up to 60 days.

### 4.3. Light-Responsive Delivery Systems

**Figure 7:**
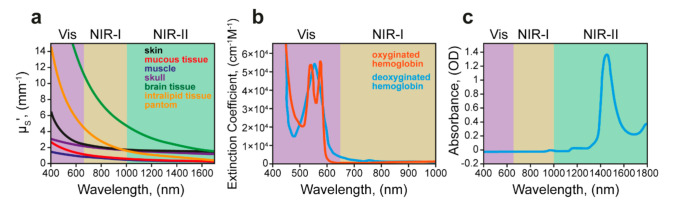
(**a**) The reduced scattering coefficient, μ_s_^′^, in the range of 400–1700 nm for various tissue types including the skin (black), the mucous tissue (red), muscle (blue), skull (violet), the brain tissue (green), and the tissue phantom, Intralipid (orange). The plots for skin, mucous tissue, muscle, and skull are derived from human samples, while that for the brain is derived from mouse samples. (**b**) Absorption spectra of oxygenated hemoglobin (orange) and deoxygenated hemoglobin (blue) in the range of 400–1000 nm, showing minimum absorbance beyond 650 nm. (**c**) The absorption spectrum of water (H_2_O) in a cuvette with a 1 mm path length, featuring strong vibrational overtone absorption bands in the 1400–1500 nm region and the >1700 nm region. Adapted with permission from [148]. Copyright 2015, American Chemical Society.

Originally, light is the most important external stimuli for all living organisms. Therefore, light exposure can be considered as a natural trigger to manipulate the distribution of delivery systems and trigger drug release. However, the light penetration in tissues may be limited by light–tissue interactions, including interface reflection, in-tissue scattering, in-tissue absorption, and tissue autofluorescence [142]. The maximum tissue transparency occurs in the near-infrared band (NIR), namely in 650–900 nm (NIR light window I) and 1100–1400 nm (NIR light window II) ranges [143]. Conventionally, the NIR I window is considered as a transparency window of biological tissues due to reduced light scattering, limited adsorption by endogenous dyes (such as hemoglobin) and water, and lower autofluorescence comparing to the light of the visible spectrum (Figure 7). Therefore, the NIR I window tends to be used for fluorescent imaging with organic dyes and inorganic nanoparticles as contrast agents in preclinical and clinical practice [144]. The penetration depth in the tissues of NIR I light is about 3–5 mm. In turn, NIR II light has even better penetration ability (9–18 mm) related to further reduced scattering, absorption, and tissue fluorescence. Therefore, many efforts are being made to develop biocompatible NIR II light-responsive agents for imaging and therapy applications [145,146,147].

**Figure 8:**
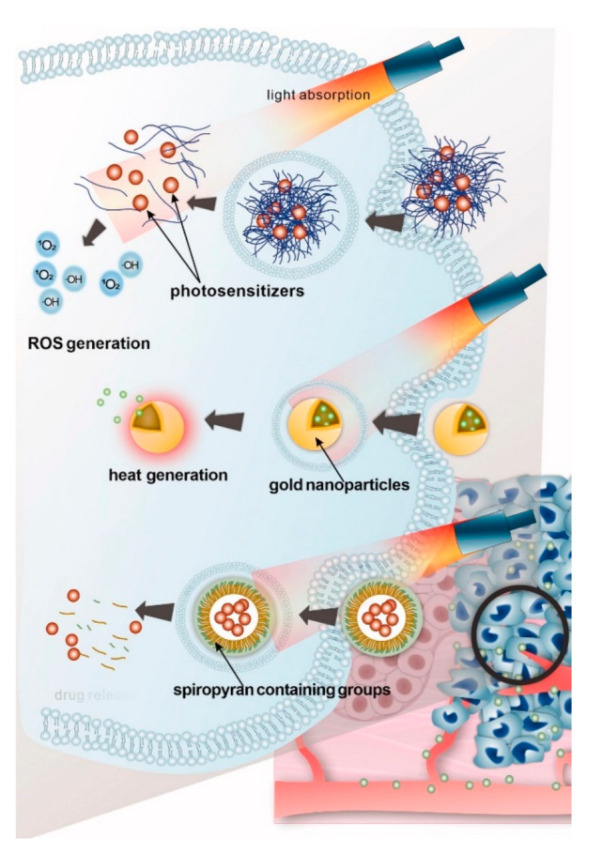
Schematic illustration of light-responsive drug delivery systems including smart delivery systems employing photosensitizers for photodynamic therapy (PDT), delivery systems employing heat mediators such as gold nanoparticles for photothermal therapy (PTT), and directly light-triggered release delivery systems. Reproduced with permission from [151]. Copyright Informa UK Limited.

The implication of light-responsive delivery systems is based on the photodynamic or photothermal therapy approaches, which means the utilization of the light energy to generate the reactive oxygen species or induce hyperthermia [149,150] (Figure 8). Furthermore, light-induced hyperthermia can be used to trigger the drug release from delivery carriers in case they are modified with the proper heat mediator [151]. The gold (Au) nanoparticles are often employed as they are biocompatible and able to convert the delivered light energy to heat with high efficiency due to the surface plasmon resonance. Additionally, the plasmon resonance frequency of Au nanostructures can be tuned in a wide range, including the NIR band, by variation of their size, shape, and aspect ratio [152,153]. Cyanine dyes, squaraine derivatives, phthalocyanine and porphyrin derivatives, and BODIPY [154] are most often chosen as NIR-responsive agents. They have absorption and emission peaks in different regions of the spectrum and are also hydrophobic or hydrophilic, which determines the areas of their use. However, other forms of active substances are also often used for NIR cancer treatment. Black phosphorus hydrogel has been used to treat breast and melanoma cancers, resulting in a shrinking tumor with minimal side effects [155]. Nano- and micromotors, which are gaining popularity in the development of drug delivery vehicles, can also be driven by light, converting the light energy into mechanical impact [156].

Probably, the main benefit of light-responsive delivery systems is the possibility of high-precision spatial and temporal control of light-induced stimuli. Modern lasers and laser diodes, which are generally used as light sources, allow for controlling the size of the light spot and the output power along with the length and repetition frequency of the light pulses. Currently, NIR lasers and diodes find wide biomedical applications including cosmetology and surgery. According to ICNIRP guidelines, the laser-induced biological effects are the result of competing mechanisms, which are photochemical, thermal, thermo-acoustic, and optoelectric breakdown, varying depending on the spectral range and exposure time [157]. Therefore, the exposure limits of laser radiation are also defined by the wavelength, exposure (pulse) duration, and the laser spot size. For instance, the skin exposure limits in the visible-NIR range (400–1400 nm) depending on the wavelength and exposure time are listed in Table 2.

In cosmetology, for most types of superficial NIR laser treatment, the radiant exposure is limited to 5–15 J∙cm^−2^. The treatment of vascular conditions requires up to 40 J∙cm^−2^. The ablative skin resurfacing is carried out at 150 J∙cm^−2^. The laser lipolysis requires the accumulation of the energy of hundreds to thousands of joules in the treated area [158].

To conclude, we refer to some recent examples of successful cancer therapy by the systemic administration of various DDS responsive to NIR light and employing PTT directly for tumor treatment or to trigger drug release. The common trend is that the systems combining chemotherapy and PTT appear more efficient than protocols employing a single treatment. Niu et al. described DDS combining PTT and chemotherapy approaches in a single formulation [159]. In particular, they employed hollow mesoporous silica nanoparticles selectively modified with chitosan conjugated with thioglycolic acid within the cavity. The modified nanoparticles were loaded with DOX and sealed with CuS nanodots. The drug release strategy is based on the disruption of disulfide bonds by glutathione in cancer cells that results in the liberation of CuS nanodots and DOX release. The NIR irradiation accelerates this effect due to PTT heating mediated by CuS nanodots. The experiments in vivo were performed with MDA-MB-231 (human breast adenocarcinoma) tumor-bearing mice by the intravenous injection of DDS into the tail vein every other day for 30 days. Additionally, a group of animals was treated with an 808 nm NIR I laser (1 W/cm^2^) for 10 min every second day (24 h after DDS injection). The proposed treatment protocol resulted in significant tumor volume reduction, while the chemo/PTT-treated mice demonstrated 60% survivability comparing to the control groups.

Amatya et al. studied the photothermal activity of iron oxide nanoparticles modified with PEG and starch to prevent aggregation and improve biocompatibility [160]. The modified nanoparticles were employed as PTT agents to treat cancer under an 885 nm NIR I laser. The PEG-starch modified iron oxide nanoparticles were intravenously injected to U87 MG (human brain glioblastoma) xenograft tumor-bearing mice (24 mg Fe/kg), and 4 h post-administration, the tumor site was laser irradiated (0.9 W output laser power) for 10 min. Comparing to the control groups of animals, the PTT after injection of PEG–starch modified iron oxide nanoparticles demonstrated the most prominent reduction of tumor size.

Luo et al. developed the multifunctional platform for combined chemo/PTT cancer therapy based on halloysite nanotubes modified with magnetite nanoparticles and polypyrrole and loaded with DOX [161]. In this formulation, magnetite nanoparticles and polypyrrole are responsible for the PTT effect. Additionally, magnetite provides one with the option of remote-controlled DDS accumulation via navigation by a magnetic field. The experiments in vivo were carried out with 4T1 tumor-bearing mice. A permanent magnet with magnetic field induction of 0.08T was attached to the tumor site before the DDS intravenous injection. In 4 h after injection, the tumor site was irradiated with 808 nm NIR I laser (1 W/cm^2^) for 8 min. The whole treatment lasted for 15 days. The mice were injected with DDS and laser-irradiated every other day. It was shown that the designed DDS can be effectively accumulated in the tumor site by the external magnetic field. The main outcome is that the combination of chemo and PTT treatment exhibited the best antitumor effect as evidenced by measurements of tumor volume ex vivo.

Fernades et al. developed the cancer therapy platform based on perfluorohexane nanoemulsions coupled to silica-coated gold nanoparticles [162]. In this formulation, the therapeutic effect is reached due to laser-induced optical absorption followed by the evaporation of the emulsion, causing internal damage to cells. Additionally, the emulsion can be loaded with chemotherapeutic agents as shown by DOX, 5-fluorouracil, and paclitaxel. The experiments in vivo were carried out with 4T1 tumor-bearing mice. The emulsions were injected intravenously (4 mg/g), and 1.5 h after injection, the tumor site was irradiated with 680 nm NIR I laser (20 mJ/cm^2^). The proposed treatment resulted in more than 65% tumor growth inhibition on day 4 of the therapy.

### 4.4. Electric Fields in Targeted Drug Delivery

Electric fields have been used in biomedicine for a long time for therapeutic purposes and for cosmetology, as well as an emergency means of restoring the heartbeat. In cancer treatment, electrochemotherapy has shown its advantage when using the pulsed electric field (PEF) combined with the cytostatic doxorubicin on the example of Sp2/0 tumors [163]. A synergetic effect of electroporation and bleomycin preparation was also shown in the rat hepatoma treatment. The origin of the enhanced joint effect of the drug and electric field stimulation is attributed to electroporation, which means that the electric field may directly improve the permeability of the cell membrane to the drugs due to destabilization of the lipid bilayer [164]. The electric field is often used for the treatment of brain diseases as a neurostimulator and to restore muscular functionality [165]. A kind of specific biomedical application of electric field stimulation is the burn treatment when the electric current affects ion transport and improves the delivery of biologically active substances, which stimulates skin repair [166].

The devices with minimal current strength and variable voltage and frequency are the most attractive for biomedical purposes. The promising technique of electric field stimulation is based on rotating external electric fields. For the first time, the effect of rotating electric fields was shown on the healthy human red blood cells. The cells become sensitive to the electric field and can be collected in threads [167]. Remote control over the cells allows not only for assembling their clusters but also distributing them in a controlled manner, solving problems related to thrombosis, for example. Furthermore, this approach may be useful for the diagnostics of biological fluids.

The values of electric fields that are allowed for human exposure vary depending on the frequency range of these fields, as well as on the duration of exposure and area of use. Electric fields interact with matter through the electric charge carried by matter. According to ICNIRP guidelines, at low frequency (up to 100 kHz), the human body is a good conductor, and the external field generates the oscillating surface charges that induce the currents inside the body [168]. At higher frequencies (100 kHz to 300 GHz), the electric field induced in the body interacts with polar molecules such as water and charged particles such as ions. Under the induced electric field, the charge molecules rotate, whereas the particles move as an electric current. Both of these result in intermolecular and interparticle interactions, causing the release of kinetic energy as heat [115]. Additionally, the electric field may be strong enough to induce electrical nerve stimulation, and short pulse repetition may cause the dielectric rupture of biological cell membranes. However, according to Adair’s study in 1991, the external 100 kV/m electric field pulses in the air with the rise times ≤ 10 ns will not affect the cell elements such as membranes, organelles, and macromolecules at the level comparable to thermal effect and therefore cannot produce biological effects at the cellular level [169]. On the other hand, he noted that high voltage pulses of sufficiently long duration (100 keV, 1 µs) may cause biological effects comparable to thermal agitation. The particular restrictions for electromagnetic field exposure in the field frequency range from 1 Hz to 100 kHz and from 100 kHz to 300 GHz are given in the corresponding ICNIRP guidelines issued in 2010 [168] and 2020 [115], respectively.

## 5. Clinical Translation of Drug Delivery Systems: Key Parameters, Challenges, and Successful Examples

The increased research activity aiming at improved delivery of pharmaceutical molecules indicates the expansion of the field. Successful clinical and commercial translation of early-stage research ideas is critically important for the future evolution of drug delivery [85]. Novel drug delivery systems are beginning to enter clinical trials, and some have already reached the market. To accomplish successful clinical translation, the system must be safe, successfully perform its therapeutic function, provide convenient administration, and offer ease of manufacturing [85]. The drug loading, release characteristics, pharmacokinetics, and biodistribution of this system must be competitive enough to warrant its development [170]. The use of the carriers for drug transportation should be a reasonable approach to increase its bioavailability [170].

Furthermore, scalability and reproducibility are critically important for the drug delivery system. Pharmaceutical technology deals with the administration of drugs or diagnostic agents to patients whose health and physiological status differ considerably from one individual to another, thus requiring a certain level of robustness from the delivery system [170]. There is controversy regarding the applicability of microfluidics for pharmaceutical streaming to provide increased product yields. The applicability of this technique is supported by a large number of commercially available devices; success in the production of chips of various configurations, up to 3D; the ability to work with a large class of reagents; and significantly high efficiency of the installation (>90%). In addition, microfluidics can also be used when testing carriers of drugs and pure drugs, for example, when conducting drug screening, development, testing, toxicity, sensitivity, drug resistance assessment, drug metabolism, pharmacokinetics, the chiral separation of drugs, and drug interactions [171]. However, on the other hand, several limitations significantly affect the applicability of microfluidics technology for applications in the pharmaceutical industry, namely: (i) the inevitable dilution of the final drug solution with a solvent, (ii) the impossibility of implementing installations using microfluidics for the needs of gene therapy, (iii) very small size of the chip for microfluidics (in the case of the formation of nanocarriers), which excludes the use of several standard units during design, significantly increasing the cost of the final product, (iv) the impossibility of obtaining small amounts of the drug, etc. [172]. By this means, the simplicity of the engineered construction is another desirable property of the developed dosage form. Complex formulations are rarely validated by independent scientific groups, which may define their rejection.

Another important issue is that a safety profile of the novel drug delivery system should be examined with the appropriate tests in a relevant animal model. Routinely performed cytotoxicity assays do not adequately inform about the ability of intravenously injected systems to cause common side effects. It is necessary to put the idea to the in vivo tests, as a great number of systems fail during in vitro to in vivo translation [85]. Then, the excellent results obtained in animal models do not guarantee future success in humans. The selection of an appropriate animal model is critically important to obtain more comprehensive information on the system, allowing the prevention of some fall-outs at the stage of its translation to clinics.

The examples of clinically and commercially available drug delivery systems and their interesting features are comprehensively discussed in other reviews [173,174,175,176,177]. Thus, for example, in terms of anticancer treatment, compared to the plethora of successful pre-clinical studies, only 15 passively targeted nanocarriers have been approved for clinical use [178]. These systems are represented by liposomal, micellar, nanosuspension, albumin-bound, and lipid particulate formulations of paclitaxel, as well as liposomal forms of doxorubicin, daunorubicin, mifamurtide, cytarabine, and irinotecan. According to [176], analyzing the clinicaltrials.gov database (the website was accessed on 15 June 2021), by the end of 2018, a total number of 75 cancer nanomedicines were under clinical investigation. The majority of phase 3 trials listed there were again associated with liposomal, micellar, albumin-bound anticancer drugs, or polymeric conjugates [176].

According to the clinicaltrials.gov database (the website was accessed on 15 June 2021), there are several successful intravenous non-liposomal particulate formulations that are in clinical trials at the moment. For instance, a tracer for malignant brain tumor imaging based on ultrasmall silica particles is currently going through a phase I clinical trials (Identifier: NCT03465618). There is also a phase 2 clinical trial studying the use of targeted silica nanoparticles for real-time image-guided intraoperative mapping of nodal metastases in patients with head and neck melanoma, colorectal, and breast cancers (Identifier: NCT02106598). Calcium carbonate is also considered a promising drug carrier. To date, there are 69 completed clinical studies with formulated conclusions including the employment of calcium carbonate at one of the stages of treatment. Although calcium carbonate is most commonly used as a dietary supplement, clinical studies have shown that osteoporosis, cancer, pain syndromes of various etiologies, sclerosis, and liver diseases also include calcium carbonate therapy in various doses.

## 6. The Development of Targeted Drug Delivery Systems from the Design to Clinical Trials

**Figure 9:**
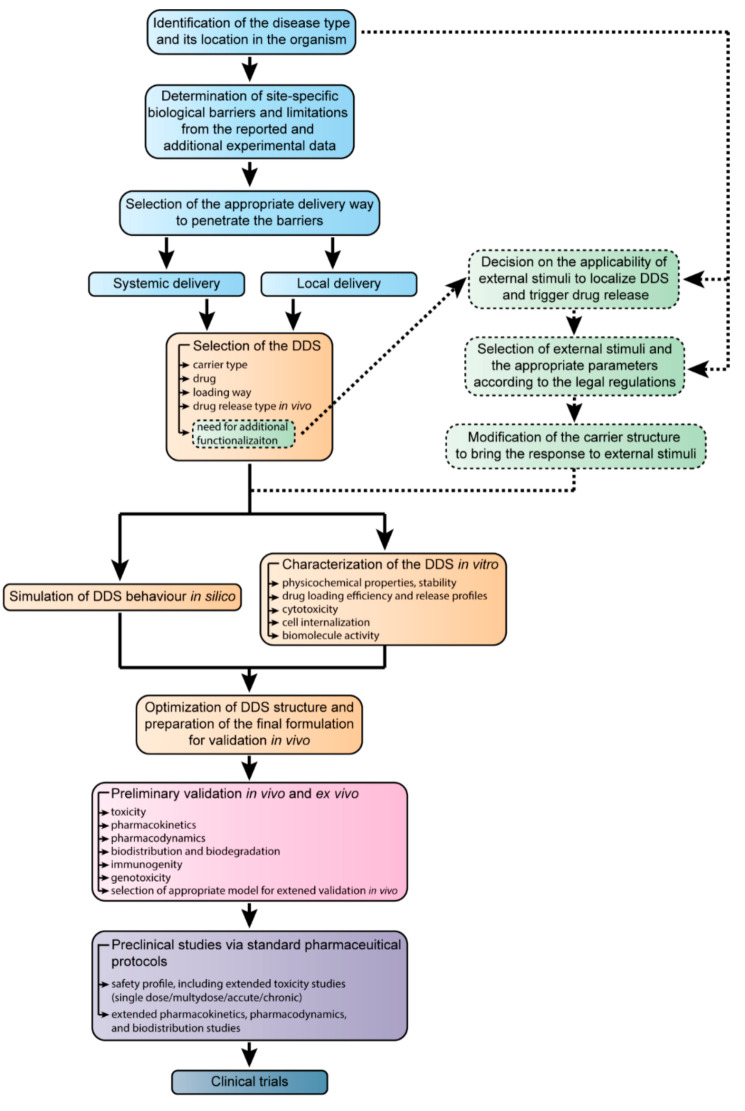
The schematic representation of the development of novel drug formulations for targeted delivery from the identification of the disease to clinical trials. The abbreviation DDS corresponds to the drug delivery system.

The attempts to figure out a “roadmap” for developing novel drug formulations from their emerging demand to the final clinical translation were taken by many researchers and product managers [179,180,181]. In the final part of this review, we have summarized the most essential steps in the development of drug delivery systems and propose our view of these steps that are shown in Figure 9.

The proposed scheme describes the development process for the narrow-spectrum drug formulations implied for the treatment of the particular disease that can be intimately detected by modern diagnostics, theranostics, and simulation methods [182,183,184]. In the case of the broad-spectrum drugs, at the first step, one has to consider the possible range of diseases, their location in the organism, and the mutual behavior of the pathologies, which is much more complicated.

Before the drug delivery, the site-specific barriers and other obstacles that may impede its efficacy have to be defined [185,186,187]. This can be performed based on the results of the functional and other types of diagnostics along with the already available data on the disease. In modern clinical practice, a medical consensus of the experts makes the diagnosis and prescribes the therapeutic regimen and/or surgical treatment [188,189]. In the development of the drug delivery systems, the case management team tends to be substituted with the data available in research papers, patents, books, and atlases of therapy of diseases and associated processes.

Considering the general strategy of the pharmacological treatment, the researchers distinguish systemic and local ways of drug delivery [190,191,192,193]. These imply the various routes of the targeted delivery system administration such as oral, intravenous, intraarterial, parenteral, transdermal, and some others. In the proposed scheme, the terms systemic and local delivery mean strategies of the treatment rather than the particular ways of drug administration.

The delivered drugs may be lipophilic or hydrophilic, with high or low solubility in the solvent medium and biological fluids. Alternatively, the drugs can be classified for their pharmacological class, which may be cytostatics, vitamins, hormones, and antibiotics, or action sites, such as cardiovascular or gastrointestinal drugs (see Table 1). Moreover, the novel therapeutics types include nucleic acids, peptides, proteins, and cells emerging alongside conventional low-molecule drugs. The loading way should be chosen carefully to preserve the structure and activity of the drug, as some substances are not suitable for heating, sonication, microwave treatment, or pH alteration [194,195,196]. Additionally, at this step, the drug release profile should be taken into account. This may be either immediate release- or delayed-release formulations. Finally, the additional delivery system functionalization has to be considered.

Functionalization allows for relating the efficiency of treatment with the external stimuli employed in clinical practices, and it is suitable to trigger the drug release and control the delivery system localization [197,198,199,200]. The decision on the applicability of the additional external stimuli should be made concerning the disease location. For instance, patients with cardiovascular implants are unable to undergo therapy involving whole-body exposure to electric or magnetic fields. The threshold of external exposure should be chosen according to the legal regulations comprising the exposure rates for the particular parts of the body. Eventually, the drug delivery system has to be modified in a way to provide an adequate response under allowed exposure rates.

The simulation of delivery system behavior in silico becomes a more and more versatile tool regarding the development of new “green” synthesis technologies [201,202]. Currently, the neural networks take into account many years of research and medical practice to simplify the experimental study and allow for faster translating of novel pharmaceutical products to clinical practice [203].

The study of delivery system properties in experiments in vitro is an intrinsic part of drug delivery development. The main ways of pharmaceutics translation from experiments in vitro to models in vivo are discussed in Section 5.

The results of computer simulations and preliminary experiments lead to the optimized drug delivery system formulation for further validation in vivo. The optimization may include additional delivery system modification to reduce its recognition by the immune system, assessment of doses and drug release profiles in the real biological systems, elaboration of the thresholds of the external stimuli exposure.

The preliminary testing of the drug delivery systems on animal models must be carried out according to ethics committee regulations. Typically, testing in vivo involves several stages, which depend on the particular pharmacological models [204,205,206]. Figure 9 specifies the most common parameters of animal model testing such as toxicity, pharmacokinetics, and pharmacodynamics that are basic in most of the protocols.

The preclinical studies are carried out in special institutions. The results of these studies are summarized in the protocols that are used as a basis for further clinical trials. The preclinical studies are required for in-life probing of the novel drug formulations. The obtained data on the safety profiles, toxicity, and biodistribution are essential for understanding if the developed drug formulations meet the safety requirements and preserve their therapeutic effect with the highest possible “benefit-to-harm” ratio [207,208].

The clinical trials are the final point of our scheme since, at this stage, the developed drugs keep their formulation, while the main subject of the study is the treatment protocols [209,210,211]. On top of that, clinical trials are a complicated topic that can be considered as the subject of a separate review.

## 7. Conclusions

At its current state of the art, the development of novel drug formulations for controllable and targeted delivery goes far beyond the laboratory-scale experiments on the encapsulation or conjugation of drugs with various carriers. Many of these novel formulations aim to beat the particular disease, and, therefore, should be designed considering its clinical course and treatment protocols, associated biological barriers and limitations, and delivery routes. Moreover, the emerging novel types of pharmaceutics require the development of specific ways of targeted delivery. Additionally, various external stimuli are approved for the clinical practice and, thus, can be used to improve the site-specific localization of delivery systems and control the drug release under conditions given in the legal regulations.

Thus, the successful translation of the novel drug formulation from the design to clinical trials demands a complex approach taking into account all of these factors and many others. This gives rise to computer simulations becoming an emerging trend in the development of delivery systems to accomplish and simplify the experimental work.

Probably, the most sufficient challenge in targeted drug delivery is overcoming the biological barriers. Thus, the delivery system has to be initially designed in a way providing it with the most efficient penetration ability. This can be achieved through the choice of the appropriate drug carrier type of particular shape, charge, and surface properties giving the desired lifetime and preventing non-specific localization. Furthermore, these may be enhanced by additional modification of the drug carriers with functional agents to bring the modality of remote control by external stimuli or by modification with special ligands, antibodies, and vectors to improve the passive targeting [212].

Currently, the drug delivery systems based on nanoparticles, polymers, capsules, and hydrogels aim for the same goal in drug therapy, namely, to reduce or eliminate the side effects while maintaining the therapeutic efficacy [213]. The different diseases require different drug delivery profiles and, therefore, the “benefit-to-harm” ratio becomes an important option for validation of the drug delivery formulations.

The proposed “roadmap” is an attempt to arrange the complex research data on the development of effective drug formulations for targeted delivery with eventual translation to clinical trials. As a complex approach, the “roadmap” and its single steps may be extended and elaborated following the state of the art in controlled drug delivery.

We believe that the employment of more or less unified approaches in the development and promotion of drug delivery systems along with the translation of the experience of their successful implementation in clinical practice will help to build the versatile industry of personalized drug formulations to meet the demands of modern medicine.

## Figures and Tables

**Table 1:** Types of drug encapsulation systems, their targeted delivery, and payload release after their intravascular administration in vivo.

Type of Drug	Drug	Core/Shell	Targeting/Release	In Vivo Evaluation	Ref
Anticancer	Paclitaxel, Doxorubicin (DOX)	-/Exosomes released by macrophages	Exosome fusion with target cells occurs more efficiently under acidic conditions, implying that exosomes may be taken up preferentially by tumors/-	Carriers with loaded drugs demonstrated superior inhibition of pulmonary metastases growth in the Lewis lung cancer (LLC) mouse model. Three mechanisms have a significant impact on carriers with loaded drug anticancer activity, i.e., (1) preferential accumulation in cancer cells, (2) efficient delivery of incorporated cargo into target cancer cells, and (3) bypassing Pgp-mediated drug efflux in resistant cancer cells.	[34]
	DOX	-/Exosomes released by immature dendritic cells (imDCs)	By exosomal membrane protein/-	DOX delivered by the carrier slows tumor growth four-fold without overt toxicity.	[35]
	DOX	-/A33 antibody-functionalized exosomes released by LIM1215 cells	By exosomal membrane protein/-	The mean tumor treated by exosomes with A33 antibodies was 3.04- and 2.90-fold lower than in the control DOX and exosomes without antibodies groups on day 16.	[36]
	Glycyrrhizin and DOX	-/Alginate nanogel particles	Specific binding of glycyrrhizin and glycyrrhetinic acid GL with cellular membranes of hepatocytes (liver targeting)/-	After 14 days of the experiment, the particles with glycyrrhizin and DOX could inhibit the growth activity of tumor cells and promote apoptotic of tumor cells to enhance antitumor effects.	[37]
	DOX loaded to Tween 80 micelles	Tween 80 micelles/Silica nanoparticles	-/-	On the 18th day, the tumor size of the SiNPs/DOX group was two-fold smaller than that of the free DOX group and four-fold smaller than that of the PBS group.	[38]
	DOX	Mesoporous silica nanoparticles/Peptide-BSA-LA	-/In the presence of metalloproteinases	After 20 days of the experiment, the control tumor was 6.7 times larger than the encapsulated drug-treated tumor.	[39]
	DOX and Mn^2+^-chelated chlorin e6 (Ce6(Mn))	CaCO_3_/PEG	-/Highly sensitive to reduced pH	Tumor growth on mice treated by carriers with the loaded drug was greatly inhibited after combined photodynamic & chemotherapy, demonstrating the superior synergistic antitumor effect by those two kinds of therapies.	[40]
	DOX	CaCO_3_/poly(acrylic acid)	-/pH-sensitive	Carriers with the loaded drug showed significantly higher tumor suppression than free drugs due to the enhanced permeability and retention effect and the pH responsiveness of NPs.	[41]
	BACE1 siRNA	-/Rabies viral glycoprotein (RVG) exosomes	Targeting was achieved by engineering the dendritic cells to express Lamp2b/-	Three days after administration and a significant protein knockdown in both siRNA-RVG-9R-treated and siRNA-RVG exosome-treated mice was observed, resulting from a significant decrease in BACE1 mRNA levels.	[42]
	KRASG12D siRNA	-/Exosomes derived from normal fibroblast-like mesenchymal cells	-/-	Diminished pancreas desmoplasia, enhanced cancer cell apoptosis, suppressed cancer cell proliferation, reduced phospho-ERK, phospho-AKT, and Kras levels are noted in KTC tumors, as well as diminished oncogenic KrasG12D expression with iExosomes treatment.	[43]
Nucleic acid	Anti-EGFR siRNA	rPAA-Chol polymer/Cationic lipid	-/-	All mice treated with siRNA formulations did not show significantly increased IFN-α and IL-6 levels compared with the 5% glucose group. These results demonstrated that LP/siRNA NPs can silence specific gene without arising innate immune responses in vivo. Carriers promising delivery systems for siRNA application in cancer treatment.	[44]
	Let-7	-/Exosomes released by HEK293 cells	GE11 at membrane/-	Exosomes delivered let-7a potently inhibited the expression of HMGA2 mRNA in A549 lung adenocarcinoma cells. Exosomes delivered let-7a inhibits tumor development via previously unidentified or uncharacterized genes in HCC70 breast cancer cells.	[45]
	Fluorescently labeled FAM-siRNA	Calcium phosphate/PEGylated carboxymethyl chitosan	-/pH-depending release	On day 14, the tumor volume in mice treated with the sihTERT nanoparticles was around 43.5% of the average volume of the PBS group. This indicated that sihTERT, the delivery of which was mediated by NPPEG-CMCS/CaP, was responsible for tumor growth suppression.	[46]
	Cas9/sgRNA	-/Exosomes released by SKOV-3 cells	-/-	Taken together, CRISPR/Cas9-loaded exosomes administered intravenously or intratumorally could deliver PARP-1 to tumor sites, causing anticancer effects. The tumor volume decreases 2.7 times after 20 days.	[47]
	PHD2	-/Poly[DMAEMA-b-(BMA-co-PAA-co-DMAEMA)]	-/pH-depending release	PHD2-NPs increased both the number and size of vessels within the scaffolds. PHD2-NPs increased the vascular volume by 300% and increased the mean vascular thickness by 137%.	[48]
	Anti-luciferase gene	-/PEG-CPB-PEI (PCPP)	-/Phenylboronic acid is targeting the SA-terminated sugar chains on cancer cells	In vivo studies demonstrated that PBA-based nanoparticles effectively accumulated in tumors and inhibited tumor growth and metastasis in the 4T1 orthotopic mammary tumor model after intravenous administration.	[49]
	RFP siRNA	-/Hyaluronic acid-*graft*-poly(dimethylaminoethyl methacrylate) (HPD) conjugate	-/Biodegradability of HA	The tumor site after 5 days was significantly reduced for the mice treated with siRNA carriers. The high tumor targetability of siRNA carriers resulted in the suppression of tumor growth, owing to its cytotoxicity against cancer cells.	[50]
	FAM-siNC, cy3-siNC, siLuc, and siBcl2	CaP/Disulfide cross-linked HA	-/Biodegradability of HA	The mass of tumors treated with loaded CaP/HA containers was only 25% of the tumor mass in the PBS group, and the tumor inhibition rate was about 80%.	[51]
	Catalase BDNF	Exosomes	-/- -/radiolabeling	Exosomes loaded with catalase efficiently accumulate in neurons and microglial cells in the brain and produce a potent neuroprotective effect. Exosomes can penetrate the vascular barrier but can make no conclusions regarding their ability to penetrate the blood–CSF barrier.	[52,53]
	Insulin	Polymethacrylic acid–polyethylene glycol–chitosan-based hydrogel microparticles	-/pH-sensitive	Within 2 h of receiving the encapsulated dose, a lowering of blood glucose level was observed in the diabetic animals.	[54]
Growth-factor	Immunoactive TLR-3 poly(I:C)	PLGA/Thiolated silica	Toll-like receptor 3/Immunostimulation effect	The therapeutic efficacy of different formulations was evaluated in the arthritic mouse. An intravenous injection of nanoparticles into mice led to a particle accumulation mainly in the lung and in the liver. The expression of IFN-α/β, TNF-α, IL-6, and IP-10 in hepatocytes, NPCs, and LSECs was significantly increased.	[55]
Other	Dexamethasone sodium phosphate	Calcium phosphate gel nanoparticles/Sialic acid-modified PEGylated lipid bilayer	E-selectin-receptors-mediated endocytosis/pH-sensitive treatment of acute kidney injury	The capsulated drug significantly improved the renal function, decreased the level of pro-inflammatory factors, and adjusted the oxidative stress factors and apoptotic proteins compared to free Dsp solution in pharmacodynamic studies. Moreover, few negative effects on blood glucose and bone mineral density were observed.	[56]
	Adapalene	PLA-PEG NP blended with low molecular weight PLA or PCL; PLGA NP/-	Receptor β (RARβ)/-	Treatment with adapalene-loaded nanoparticles was able to elicit a biological response as quickly as 4 h post injection in reporter mice, and these effects were sustained for a minimum of 24 h.	[57]

**Table 2:** Laser radiation exposure limits for the skin [157].

Exposure Limits		Correction Factor, *C_A_*	
Exposure Duration, *t*	Definition	Wavelength, *λ*	Definition
1 ns–100 ns	200∙*C_A_*, J∙m^−2^	400–700 nm	1.0
100 ns–10 s	11∙*C_A_*∙*t^0.25^*, kJ∙m^−2^	700–1050 nm	10^0.002(*λ*/1–700)^
10 s–30 ks	2.0∙*C_A_*, kW∙m^−2^	1050–1400 nm	5.0
